# Aberrant Expression of Human Endogenous Retroviruses and *SETDB1* in Adolescents with Anorexia Nervosa

**DOI:** 10.3390/ijms27093755

**Published:** 2026-04-23

**Authors:** Federico Amianto, Pier-Angelo Tovo, Alice Po, Cristina Calvi, Chiara Davico, Paola Montanari, Elena Rainò, Antonella Anichini, Serena Vesco, Daniela Bechis, Cristina Marotta, Stefano Gambarino, Ilaria Galliano, Massimiliano Bergallo

**Affiliations:** 1 Neurosciences Department, University of Torino, Via Cherasco 15, 10126 Turin, Italy; federico.amianto@unito.it; 2 Department of Pathology and Care of the Child, AOU Città della Salute e della Scienza di Torino, Ospedale Infantile Regina Margherita (OIRM), 10126 Turin, Italy; alice.po@edu.unito.it (A.P.); cristina.calvi@unito.it (C.C.); chiara.davico@unito.it (C.D.); paola.montanari@unito.it (P.M.); elena.raino@unito.it (E.R.); antonella.anichini@unito.it (A.A.); sevesco@cittadellasalute.to.it (S.V.); daniale.bechis@unito.it (D.B.); cmarotta@cittadellasalute.to.it (C.M.); ilaria.galliano@unito.it (I.G.); massimiliano.bergallo@unito.it (M.B.); 3 Department of Public Health and Pediatric Sciences, University of Turin, Regina Margherita Children’s Hospital, 10126 Turin, Italy; stefano.gambarino@unito.it

**Keywords:** anorexia nervosa, human endogenous retroviruses (HERVs), *TRIM28*, *SETDB1*, inflammation, epigenetics

## Abstract

Human endogenous retroviruses (HERVs) represent 8% of our genome. They are remnants of ancient infections of germinal cells. HERVs are no longer infectious, but their enhanced expression is implicated in several diseases, including neuropsychiatric disorders. Their transcription is regulated by *TRIM28* and *SETDB1*, which are involved in the regulation of epigenetic processes, in neural cell differentiation, and brain inflammation. We explored the expressions of HERVs and *TRIM28*/*SETDB1* in adolescents affected by anorexia nervosa (AN). Through real-time PCR, we assessed the transcription levels of *pol* genes of HERV-H, -K, and -W, of *env* genes of Syncytin 1 (SYN1), Syncytin 2 (SYN2), and of HERV-W, and of *TRIM28* and *SETDB1* in whole blood of 37 adolescents with AN and in healthy controls (HCs) of comparable age. *HERV-H-pol*, *HERV-K-pol* and *SETDB1* transcriptional levels were significantly higher in adolescents with AN as compared with HCs, while HERV-W-*pol* and -*env* were downregulated in the former. No differences were observed for SYN1, SYN2, and *TRIM28* between the two groups. The observed expression pattern of HERVs is specific for AN as compared to other neuropsychiatric disorders. These aberrant expressions suggest a potential role of retroviral elements in the pathophysiology of AN, opening the way for innovative diagnostic and therapeutic strategies.

## 1. Introduction

Anorexia Nervosa (AN) is a severe psychiatric disorder characterized by significantly low body weight, intense fear of weight gain, and disturbed body perception [1]. Its complications affect all body systems and can be fatal [1]. The etiopathogenesis of the disease remains an unsolved enigma, and this hampers the development of novel therapeutic interventions. AN has long been considered a prevalent psychological disorder, and researchers have mainly focused on psychological fragilities. Recently, greater interest has been devoted to the biological correlates of the disease, including the genetic predisposition. Twin-based heritability of AN is estimated to reach 50–60%. Genome-wide association studies showed a genetic overlap with other psychiatric disorders, identified significant loci, and revealed direct correlations with psychiatric and metabolic components [2]. Epigenetics is an emerging element through which environmental factors can modulate gene expressions without changing their fundamental structure. Epigenetic processes are implicated in the development of mental health phenotypes, including eating disorders [3]. Other biological factors are increasingly investigated. These include the underlying inflammatory status, the dysregulation of the immune system with associated autoimmune reactions, and the intestinal microbiota dysbiosis with impact on brain functions [4,5,6].

Human endogenous retroviruses (HERVs) represent about 8% of our genome [7]. They originate from ancestral infections of germinal cells of primates millions of years ago [7,8]. Given the continuous mutations during evolution, HERVs have lost the capacity to produce infectious particles [7]. Nevertheless, their retroviral structure is maintained, with three principal genes, group associated antigens (*gag*), polymerase (*pol*), and envelope (*env*), flanked between two regulatory long terminal repeats (LTRs) [7,8]. Most HERVs are inactive, but some elements are transcribed, and a few encode proteins [7]. HERVs have been co-opted for essential biological functions during intrauterine life. Two envelope proteins, syncytin 1 (SYN1) and syncytin 1 (SYN2), are crucial for placental syncytiotrophoblast formation and contribute to maternal–fetal tolerance, given their vigorous immunosuppressive properties [9,10]. Postnatally, the physiological functions of HERVs remain unclear. Among human tissues, HERV expressions are consistently high in the central nervous system [11]. A transcriptional dysregulation of HERVs has been observed in several diseases and proposed in their etiopathogenesis. Actually, HERVs can influence the transactivation of neighboring cellular genes [12]. Their mRNAs can be retro-transcribed and reintegrated into DNA, causing mutations, or, being recognized as non-self by viral receptors, they can trigger a variety of inflammatory and immune reactions [13]. Furthermore, there are mutual interactions between HERVs and intestinal microbiota [14].

HERV expression is modulated by environmental factors via epigenetic mechanisms, such as DNA methylation and heterochromatin formation by histone tail modifications [15]. *SETDB1* is a methyltransferase with high specificity for the lysine 9 residue of histone H3 [15,16]. It is recruited by *TRIM28* to form a complex with Krüppel associated box domain zinc finger proteins (KRAB-ZFPs), the largest family of transcriptional regulators in the human genome [15,17]. Both *TRIM28* and SETBD1 represent specific tags for transcriptional modulation of HERV sequences [18,19]. Additionally, they regulate the transactivation of thousands of cellular genes and are directly involved in epigenetic processes, such as the differentiation of cell lineages, the control of brain functions, and the modulation of the immune response [20,21].

Among psychiatric disorders, enhanced expressions of HERVs and *SETDB1* have been found in children with autism spectrum disorders (ASDs) [22]. Increased expression of HERV-W has been evidenced in the brain and plasma of patients with schizophrenia, with some studies indicating a correlation with inflammatory markers and severity of cognitive impairment [23,24,25]. Overexpression of *HERV-W-env* was also described in patients with bipolar disorder [25].

These biological alterations may converge on molecular pathways known to regulate HERV expression. Despite (1) the aberrant expressions of HERVs in several neuropsychiatric disturbances and their proposed etiopathogenetic role, (2) their effects on the immune system and the intestinal microbiota, (3) the HERV associations with inflammatory markers, (4) epigenetic processes, (5) metabolic stress, and (6) the implications of *TRIM28*/*SETDB1* (a) in the regulation of HERV transcription, their direct impact on (b) epigenetic mechanisms, and (c) immune response, no study has explored HERV expressions in patients affected by AN. AN is a psychiatric disorder in which body changes are more marked and possibly related both to the pathogenesis and maintenance of the disorder [1,2,3,4,5,6]. Thus, the rationale for exploring HERV expression is strong and possibly related to relevant therapeutic implications. Based on all these considerations, the aim of the current research was to assess the transcription levels of *pol* genes of HRV-H, HERV-K and HERV-W, the three most widely studied retroviral families, of the *env* genes of HERV-W (also called multiple sclerosis-associated retrovirus, MSRV), SYN1, and SYN2, as well as of *TRIM28* and *SETDB1* in whole blood from adolescents with AN and healthy controls (HCs) of comparable age.

## 2. Results

### 2.1. Study Populations 

Thirty-seven adolescents with AN were enrolled in the study; their characteristics are reported in Table 1.

### 2.2. Transcription Levels of pol Genes of HERV-H and HERV-K in Whole Blood of Patients with AN and HCs

**Figure 1:**
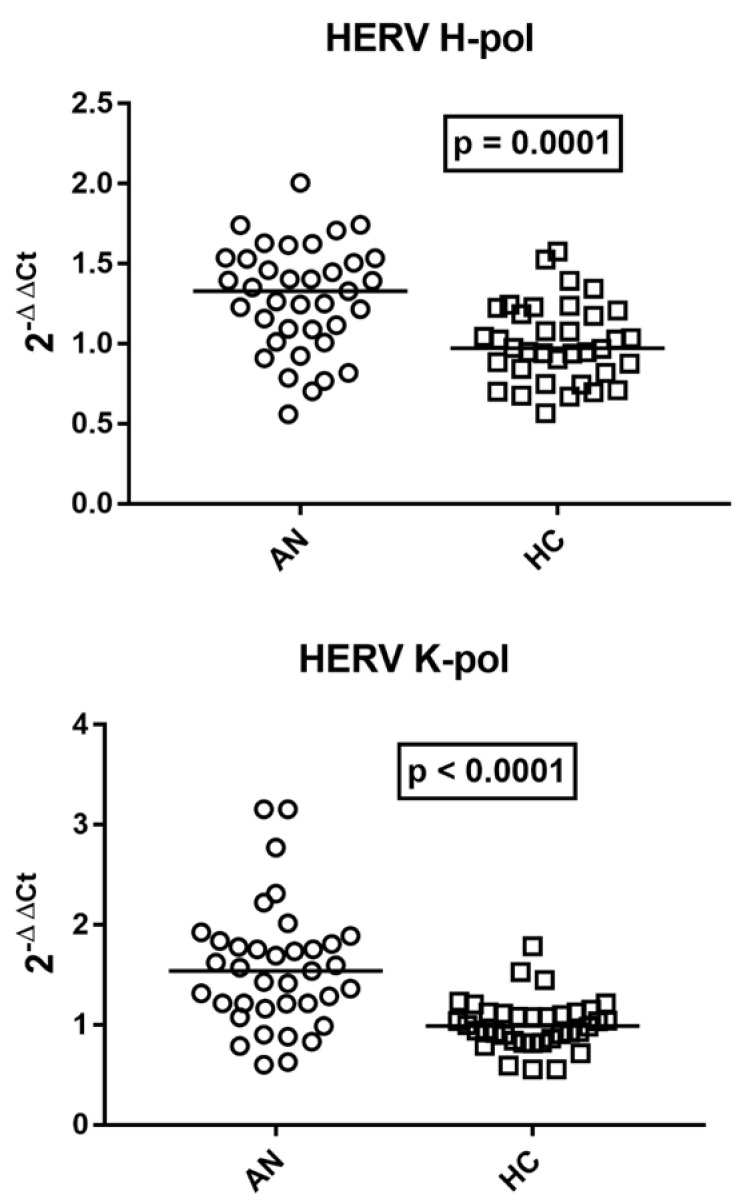
Transcription levels of *pol* genes of HERV-H and HERV-K in whole blood from 37 adolescents with AN and 36 age-matched HCs. Medians and IQR 25–75%: *HERV-H-pol*: AN 1.33, 1.09–1.53; HC 0.97, 0.84–1.21; *HERV-K-pol*: AN 1.54, 1.21–1.81; HC 1.00, 0.86–1.12. 2^−ΔΔCt^ = Relative expression according to the 2^−ΔΔCt^ method. Circles and squares show the median of three individual measurements; horizontal lines show the median values.

The transcriptional levels of *HERV-H-pol* and *HERV-K-pol* were significantly higher in adolescents with AN than in HCs (Figure 1).

### 2.3. Transcription Levels of pol Gene and env Gene of HERV-W in Whole Blood of Patients with AN and HCs

**Figure 2:**
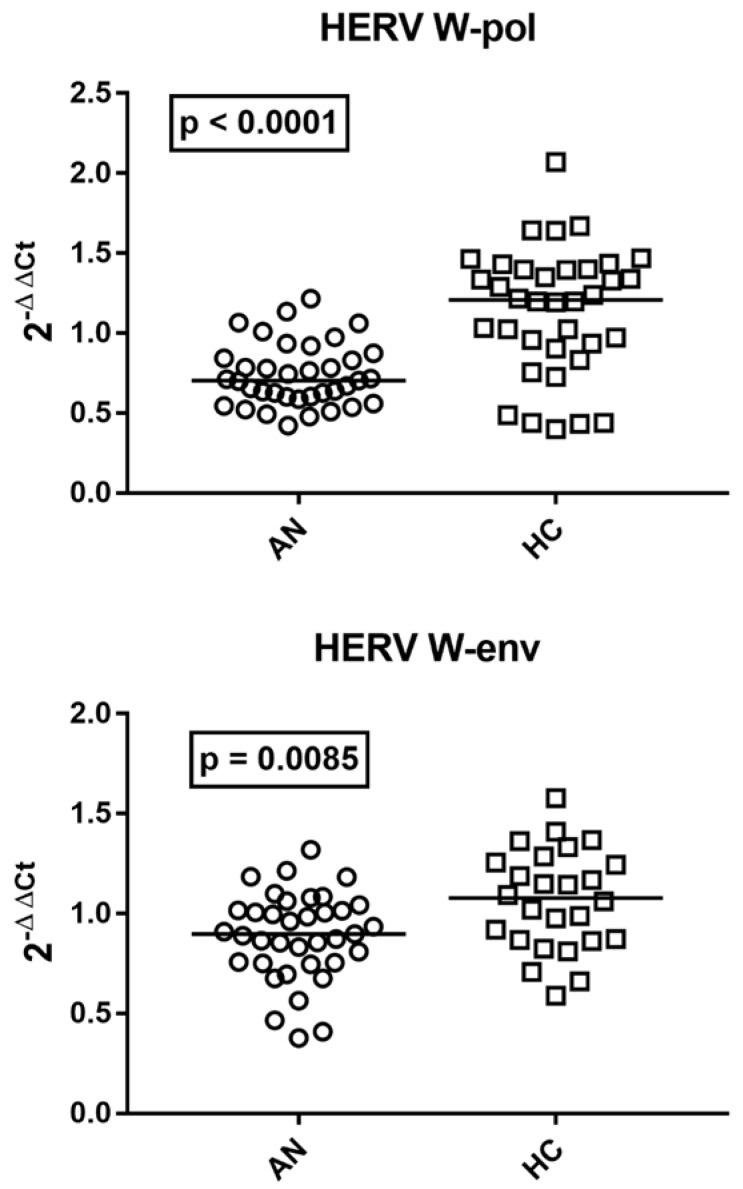
Transcription levels of *pol* gene and *env* gene of HERV-W in whole blood from 37 adolescents with AN and 36 age-matched HCs. Medians and IQR 25–75%: HERV-W-pol: AN 0.70, 0.60–0.84; HC 1.21, 0.93–1.40; *HERV-W-env*: AN 0.90, 0.75–1.02; HC 1.08, 0.87–1.25. 2^−ΔΔCt^ = Relative expression according to the 2^−ΔΔCt^ method. Circles and squares show the median of three individual measurements; horizontal lines show the median values.

The transcriptional levels of the *pol* gene and *env* gene of HERV-W were significantly lower in adolescents with AN than in HCs (Figure 2).

### 2.4. Transcription Levels of env Genes of SYN 1 and SYN 2 in Whole Blood of Patients with AN and HCs

**Figure 3:**
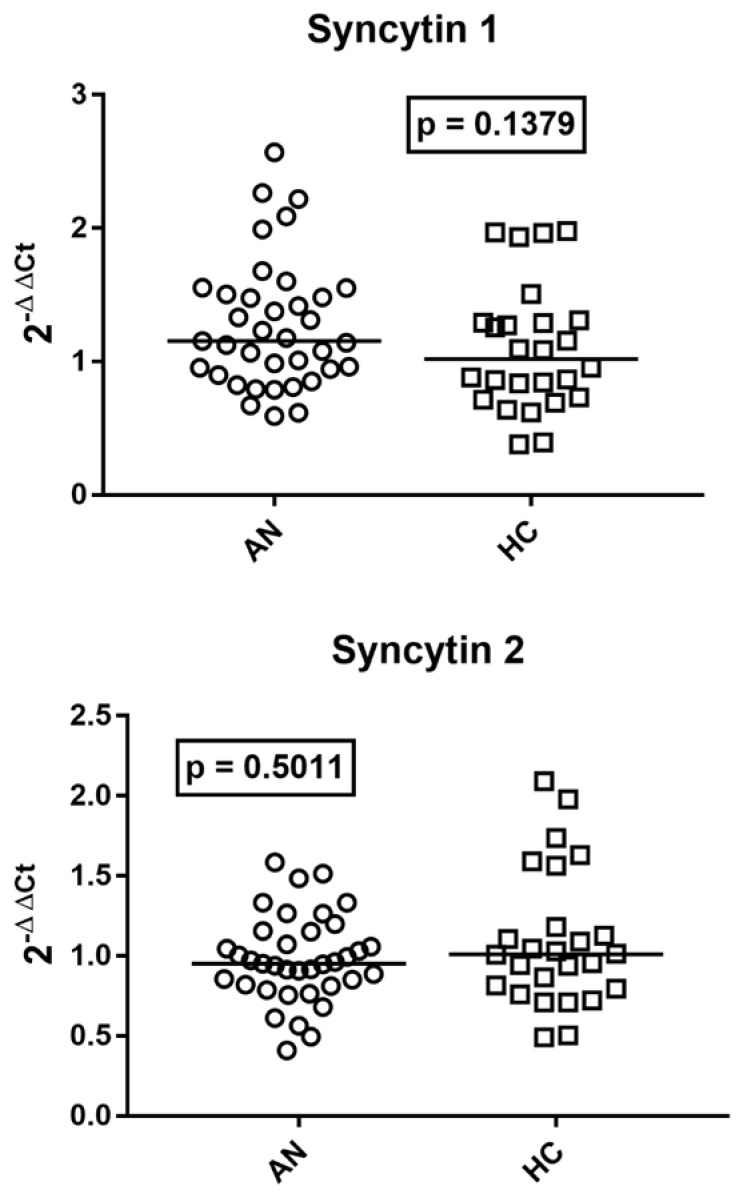
Transcription levels of *env* genes of SYN 1 and SYN 2 in whole blood from 37 subjects with AN and 26 HC. Medians and IQR 25–75%: SYN 1: AN 1.15, 0.94–1.50; HC 1.02, 0.76–1.29; SYN 2: AN 0.95, 0.82–1.15; HC 1.01, 0.80–1.17. 2^−ΔΔCt^ = Relative expression according to the 2^−ΔΔCt^ method. Circles and squares show the median of three individual measurements; horizontal lines show the median values.

The median values of RNA levels of SYN1 and SYN2 were similar in subjects with AN and HCs (Figure 3).

### 2.5. Transcription Levels of TRIM28 and SETDB1 in Patients with AN and Age-Matched HCs

**Figure 4:**
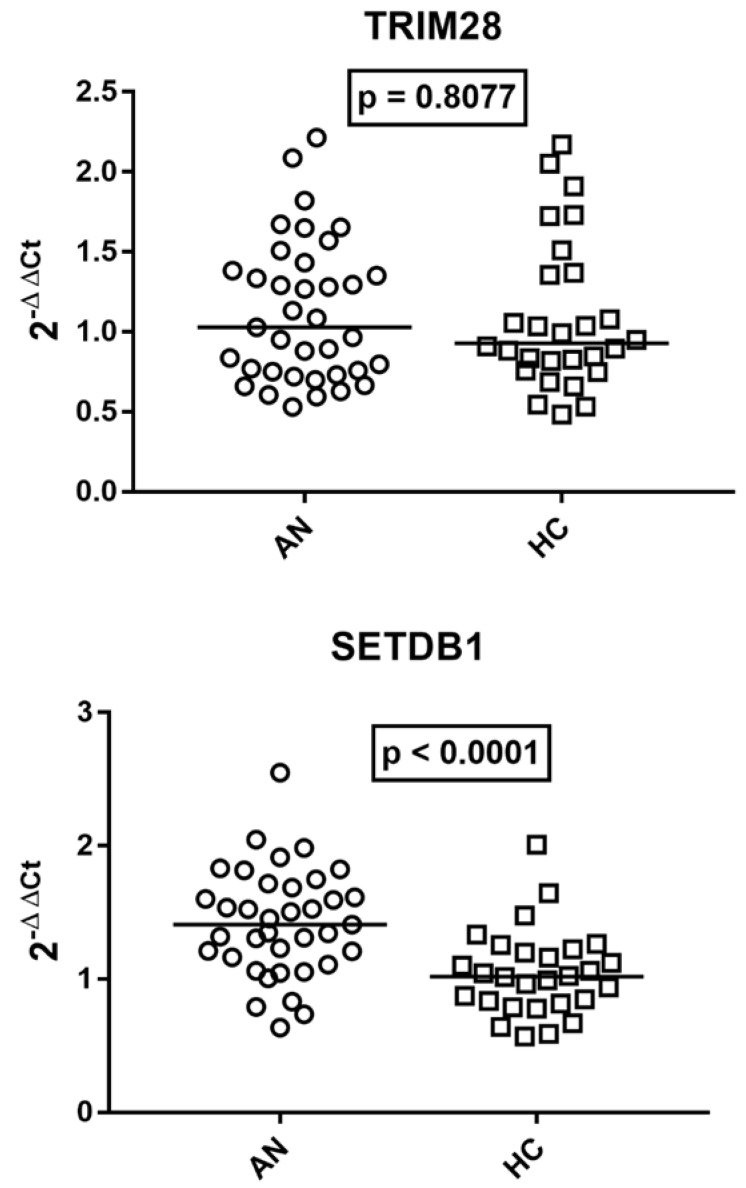
Transcription levels of *TRIM28* and *SETDB1* in whole blood from 37 adolescents with AN and 26 age-matched HCs. Medians and IQR 25–75%: *TRIM28* AN 1.03, 0.75–1.38; HC 0.93, 0.77–1.29; *SETDB1* AN 1.41, 1.16–1.69; HC 1.02, 0.84–1.19. 2^−ΔΔCt^ = Relative expression according to the 2^−ΔΔCt^ method. Circles and squares show the median of three individual measurements; horizontal lines show the median values.

As reported in Figure 4, the median mRNA levels of *TRIM28* were comparable in patients with AN vs. HCs, while the median transcriptional levels of *SETDB1* were significantly higher in AN patients than in HCs.

Effect size analysis showed large differences for *HERV-H-pol* (r = 0.51), *HERV-K-pol* (r = 0.61), HERV-W-*pol* (r = 0.60), and *SETDB1* (r = 0.53) and a moderate effect size for *HERV-W-env* (r = 0.39), while smaller or negligible effects were observed for the other targets.

Although some variability was observed within groups, particularly for *HERV-K-pol* and *SETDB1*, the overall distributions remained clearly distinct between AN patients and healthy controls.

## 3. Discussion

For the first time, our results show the pattern of expression of HERVs in adolescents with AN. We found significantly higher transcriptional levels of *HERV-H-pol* and *HERV-K-pol* as compared to HCs of comparable age. In contrast, both HERV-W-*pol* and -*env* sequences displayed reduced transcript levels, while the RNA concentrations of other retroviral elements, such as SYN1 and SYN2, were similar to HCs. It is worth mentioning that *HERV-W-env* (MSRV) and SYN-1 share a similar (but not identical) primary structure, while their proteins show distinct functions (pro-inflammatory for MSRV, syncytial and immunosuppressive for SYN1). This may account for the different concentrations of their transcripts in AN patients, as these elements derive from distinct genomic loci and may be subject to differential transcriptional regulation. The simultaneous higher and lower expression of single retroviruses in the same patients is a quite rare phenomenon. A higher prevalence of HERV-K sequences and a simultaneous reduction of HERV-W was reported also in cell-free plasma samples from patients with major burns as compared to normal individuals [26]. This impaired HERV-W expression was ascribed to the long time interval between injury and blood sample collection or to effects of therapies [26]. Actually, antipsychotic drugs can modulate HERV expression [27], and some of our patients were treated with antipsychotics. This mechanism might be shared with schizophrenic patients, but these display a different transcriptional pattern without downregulation of HERV-W-*pol* and -*env* [23,24]. Patients with bipolar disorder are also often treated with antipsychotic drugs, but they exhibit overexpression of *HERV-W-env,* not its reduction, as observed in our patients [25,28]. This supports the existence of a specific pattern of aberrant expression of retroviral sequences in adolescents suffering from AN as compared to subjects with other neuropsychiatric disorders. To this purpose it must be underlined that the differences in sample type (blood vs. brain tissue) and in age groups between our patients and the latter may limit direct comparability.

The underlying biological mechanisms responsible for the aberrant HERV expression in AN patients and their clinical meaning remain to be elucidated. Our findings suggest an apparent anomalous functioning of the interactions between HERVs and their corepressors *TRIM28*/*SETDB1*, whose activation is expected to give rise to DNA methylation and heterochromatin formation ultimately resulting in HERV silencing [19]. Given the normal expression of *TRIM28* and the enhanced transcription of *SETDB1*, the high RNA levels of *HERV-H-pol* and *HERV-K-pol* cannot be ascribed to their impaired activation. Instead, one could hypothesize that the *SETDB1* overexpression is responsible for the downregulation of HERV-W-*pol* and -*env*. It is worth mentioning that the protein complex of *TRIM28*/*SETDB1* is essential for maintaining endogenous retroviruses in a silent state in early embryos and pluripotent stem cells [18]. In contrast, when these cells differentiate into distinct somatic cells, the transcription of retroviral sequences is no longer dependent on such repressors, which sometimes may act as transcriptional activators rather than as repressors [19,29]. It cannot be overlooked that functional interactions between *TRIM28*/*SETDB1* and single HERVs could derive from post-translational events between the encoded proteins, while we assessed only their transcriptional profiles.

HERV expression is modulated by a variety of cellular stressors, including cytokine signaling, oxidative stress, and epigenetic modifications. Growing data document the increased levels of pro-inflammatory cytokines in anorexic subjects [4,5]. The release of inflammatory cytokines results in the proteasome-driven activation of NF-kB, which binds to specific motifs of HERVs that, along with inflammatory cytokines, lead to their enhanced transactivation [30]. Notably, HERVs are, in turn, able to induce and potentiate a variety of inflammatory and immune responses [7]. The final result may be a vicious circle leading to increasing reactive responses. Eating disorders and autoimmune diseases give each other a mutual increased risk [31]. This might be explained by the high transcript levels of retroviral elements in both conditions [7,27].

Abnormal composition of the gut microbiota has been reported in AN patients [6]. There are mutual interactions between intestinal microbiota and endogenous retroviruses [14]. Germ-free mice lose intestinal expression of several retroviruses, while exposure to bacteria and their products can stimulate retroviral transcription [32]. The dysbiosis of the gut microbiota in AN might thus contribute to the HERV overexpression found in our patients [6]. 

Regarding *SETDB1*, its upregulation is implicated in a large array of biological activities. It contributes to regular cellular homeostasis within the brain, while alterations have been associated with several neuropsychiatric disorders [33]. Through the complex with *TRIM28*/KRAB-ZFPs, *SETDB1* conditions B and T lineage differentiations and functions, leading to peculiar inflammatory response potentially implicated in brain malfunctioning [34].

It is worth mentioning that the overexpression of *HERV-H-pol*, *HERV-K-pol*, and *SETDB1* is consistent with that found in children with ASD [22]. This pattern of HERV transcription may represent a common biomarker of these neuropsychiatric disorders and support a shared pathogenetic role. Signs of ASD are overrepresented in individuals with AN, and those with co-occurrence of autistic traits have more severe disturbances and poorer prognosis [35,36]. The hyperactivation of the same retroviral elements and of *SETDB1* in adolescents affected by both AN and ASD may account for their more compromised mental health and worse evolution.

Differently from schizophrenia that showed an overexpression of HERV-W [23,24] and bipolar disorders where *HERV-W-env* was overexpressed [25], our findings demonstrate a reduction of HERV-W-*pol* and -*env*.

The observed downregulation of HERV-W-*pol* and -*env* in AN could derive from selective epigenetic silencing via DNA methylation or histone modifications, influenced by body mass gene regulation [37,38]. Alternatively, this could reflect a compensatory mechanism to limit excessive immune activation, particularly considering the pro-inflammatory milieu associated with AN [4].

Longitudinal studies are needed to determine whether the abnormal HERV expressions persist or disappear over the course of the eating disorder or by increasing the sample size. This could lead to the identification of useful biological markers to monitor the course of the disease. The presence of psychiatric comorbidities in the AN group may represent a potential confounding factor influencing HERV expression. Further investigations are required to explore more in depth the origin of the different HERV expression patterns observed in AN and in other mental disorders [22,23,24,25] and their potential prognostic significance, given the preliminary nature of our findings. Should HERVs be confirmed as clinically relevant in the pathogenesis or maintenance of AN, antiretroviral therapies could be explored through targeted trials to modulate HERV activity in anorexic patients unresponsive to standard treatments [39,40]. It remains, however, unclear whether reducing overexpressed HERVs or enhancing those downregulated would be beneficial.

## 4. Materials and Methods

### 4.1. Study Populations

All recruited adolescents with AN were inpatients consecutively admitted at the Division of Child and Adolescent Neuropsychiatry, University of Turin, Regina Margherita Children’s Hospital, Turin, Italy. All patients received a clinical evaluation using a semi-structured interview during the first and second visit of assessment by a trained child neuropsychiatrist, ensuring they met the DSM-5-TR diagnostic criteria for AN [41]. Inclusion criteria consisted of: 1. full diagnosis of anorexia nervosa; 2. age range 12–18. Exclusion criteria were: 1. comorbid ASD, schizophrenia, bipolar or neurologic diseases; 2. intellectual disability; 3. any active medical problems, such as infections, cancer, autoimmune disorders. HCs included asymptomatic adolescents of comparable age recruited with the same exclusion criteria who were tested at the same hospital for routine laboratory examinations and whose results were all within the normal reference range.

The parents or legal guardian of all participants signed an informed consent for the purpose of including a minor into the present trial.

### 4.2. Blood Sample Storage

HERV expression may vary across different tissues. Studying their transcription in brain tissue from anorexic patients would have been more appropriate. However, this is not feasible in this clinical context for ethical reasons, particularly when considering age-matched healthy controls. Therefore, we performed the analyses using peripheral blood samples from AN patients and HCs. Blood samples were mixed with RNAPro Solution (Biomole, Turin, Italy) to maintain RNA integrity and subsequently stored at −80 ◦C until RNA extraction. Collection, preservation, storage, and processing procedures were the same for both patients and the control group to reduce pre-analytical variability.

### 4.3. Total RNA Extraction 

Total RNA was extracted using the automated extractor Maxwell with an RNA Blood Kit (Promega, Madison, WI, USA), which includes treatment with DNase during the RNA extraction process. To further exclude any contamination of genomic DNA, RNA extracts were directly amplified without reverse transcription. RNA concentration and purity were assessed by traditional UV spectroscopy with absorbance at 260 and 280 nm (SimpliNano spectrophotometer, Biochrom US, Holliston, MA, USA). The RNAs were stored at −80 °C until use.

### 4.4. Reverse Transcription

Four hundred nanograms of total RNA was reverse-transcribed with 20 μL of buffer 10X, 4.8 μL of MgCl_2_ 25 mM, 2 μL ImpromII (Promega, Madison, WI, USA), 1 μL of RNase inhibitor 20 U/L, 0.4 μL random hexamers 250 μM (Promega, Madison, WI, USA), 2 μL mix dNTPs 100 mM (Promega, Madison, WI, USA), and dd water in a final volume of 20 μL. The reaction mix was carried out in a GeneAmp PCR system 9700 Thermal Cycle (Applied Biosystems, Foster City, CA, USA) under the following conditions: 5 min at 25 °C, 60 min at 42 °C and 15 min at 70 °C for the inactivation of enzymes; the cDNAs were stored at −20 °C until use.

### 4.5. Transcription Levels of HERVs, TRIM28 and SETDB1 by Real-Time PCR Assay

Relative expression of transcription levels of *pol* genes of HERV-H, -K and –W and of *env* genes of HERV-W (MSRV), SYN1, and SYN2, as well as of *TRIM28* and *SETDB1*, were achieved as previously described in detail using the primers and probes reported in Table 2 [42]. In particular, the use of specific primers and probes for *HERV-W-env* (MSRV) and SYN1 allowed us to assess the expression of these distinct HERV-W-derived elements. Briefly, 40 ng of cDNA wsd amplified in a 20 μL total volume reaction, containing 2.5 U goTaQ MaterMix (Promega, Madison, WI, USA), 1.25 mmol/L MgCl_2_, 500 nmol of specific primers, and 200 nmol of specific probes. All the amplifications were run in a 96-well plate at 95 °C for 10 min, followed by 45 cycles at 95 °C for 15 s and at 60 °C for 1 min. Each sample was run in triplicate. Relative expression of target gene transcripts was performed according to the 2^−ΔΔCt^ method [43]. GAPDH was selected as a reference gene, as it has been shown to have good efficiency and excellent reproducibility with constant expression in human leukocyte samples and previously used in our studies [44]. After normalization of the PCR result of each target gene with the housekeeping gene, the method includes additional calibration of this value with the median expression of the same gene evaluated in a pool of healthy controls. The results, obtained with the 2^−ΔΔCt^ method, show the variations in target gene transcripts relative to the standard set of controls. Since we measured Ct for every target in all samples, we argue that our methods were suitable for HERVs, *SETDB1* and *TRIM28* detection and quantifications. Ct values of the housekeeping gene GAPDH were comparable between AN patients and healthy controls (*p* = 0.1916), supporting similar RNA quality and amplification efficiency across groups.

### 4.6. Statistical Analysis

The Mann–Whitney test was used to compare the relative transcription levels of pol genes of HERV-H, -K and -W, of env genes of SYN1, SYN2 and HERV-W, and of *TRIM28* and *SETDB1* between patients with AN and healthy controls. Effect sizes for Mann–Whitney comparisons were estimated using rank-biserial correlation. Statistical analyses were done using Prism software (GraphPad Software, Version 7, San Diego, CA, USA). In all analyses, *p* < 0.05 was taken to be statistically significant.

## 5. Conclusions

In conclusion, our results show a specific pattern of aberrant expression of HERVs in AN. This might represent a molecular feature of the disorder implicated in its pathophysiology. Our findings might provide insights towards novel treatment strategies, though longitudinal investigations are needed to assess the evolution of these biological markers in different phases of disease and their possible correlation with therapeutic treatments. Limitations of our study include the simple design, based only on qPCR assays, the limited sample size and the lack of formal RNA integrity assessment (e.g., RIN values). We did not evaluate HERV protein translation, immune response to retroviral antigens, or correlations with the pro-inflammatory cytokine profile. Nevertheless, our research may pave the way for future targeted investigations, being the first time that HERVs and *TRIM28*/*SETDB1* have been explored in AN. The age of our patients seems to suggest that the alterations found are already present in a relatively early stage of the disease. The increase in *SETDB1* activity is in line with the variations in epigenetic processes observed in AN, possibly suggesting their metabolic origin. The different HERV expressions in patients with AN highlight novel host–genome interactions, potentially influenced by factors such as food restriction, metabolic distress, and pro-inflammation mechanisms. Our findings may open new research avenues, such as the discovery of useful AN biomarkers, innovative therapeutic trials in patients unresponsive to standard treatments, and the exploration of AN biological risk factors. 

## Figures and Tables

**Table 1:** Demographics and clinical characteristics of the sample. IQR: inter-quartile range. * Comorbidities: at least one among major depression, polarized anxiety disorder, obsessive-compulsive disorder or post-traumatic stress disorder. ** Pharmacotherapy: at least one among SSRI, benzodiazepines or atypical antipsychotics.

Total sample, n	37
Females, n (%)	37 (100)
AN-R, n (%)	37 (100)
DH service, n (%)	17 (46)
Inpatient, n (%)	20 (54)
Age, yr, median (IQR)	14.95 (14.23–15.96)
Age at onset, yr, median (IQR)	13 (12–14)
Age at admission, yr, median (IQR)	14 (13–15.5)
Comorbidities *, n (%)	37 (100)
Pharmacotherapy **, n (%)	31 (83.78)
Bingeing, n (%)	1 (2.70)
Vomiting, n (%)	3 (8.10)
Misuse of laxatives, n (%)	1 (2.70)

**Table 2:** Primers and probes used to assess the transcription levels of *pol* genes of HERV-H, -K and –W, of *env* genes of Syncytin 1, Syncytin 2 and HERV-W (MSRV), of *TRIM28* and *SETDB1*, and of *GDPH*.

Name	Primer/ Probe	Sequence
*HERV-H pol*	Forward	5′-TGGACTGTGCTGCCGCAA-3′
	Reverse	5′-GAAGSTCATCAATATATTGAATAAGGTGAGA-3′
	Probe	6FAM-5′-TTCAGGGACAGCCCTCGTTACTTCAGCCAAGCTC-3′-TAMRA
*HERV-K pol*	Forward	5′-CCACTGTAGAGCCTCCTAAACCC-3′
	Reverse	5′-TTGGTAGCGGCCACTGATTT-3′
	Probe	6FAM-5′-CCCACACCGGTTTTTCTGTTTTCCAAGTTAA-3′-TAMRA
*HERV-W pol*	Forward	5′-ACMTGGAYKRTYTTRCCCCAA-3′
	Reverse	5′-GTAAATCATCCACMTAYYGAAGGAYMA-3′
	Probe	6FAM-5′-TYAGGGATAGCCCYCATCTRTTTGGYCAGGCA-3′-TAMRA
*Syncytin 1 env*	Forward	5′-ACTTTGTCTCTTCCAGAATCG-3′
	Reverse	5′-GCGGTAGATCTTAGTCTTGG-3′
	Probe	6FAM-5′-TGCATCTTGGGCTCCAT-3′-TAMRA
*Syncytin 2 env*	Forward	5′-GCCTGCAAATAGTCTTCTTT-3′
	Reverse	5′-ATAGGGGCTATTCCCATTAG-3′
	Probe	6FAM-5′-TGATATCCGCCAGAAACCTCCC-3′-TAMRA
*HERV-W env*	Forward	5′-CTTCCAGAATTGAAGCTGTAAAGC-3′
	Reverse	5′-GGGTTGTGCAGTTGAGATTTCC-3′
	Probe	6FAM-5′-TTCTTCAAATGGAGCCCCAGATGCAG-3′-TAMRA
*TRIM28*	Forward	5′-GCCTCTGTGTGAGACCTGTGTAGA-3′
	Reverse	5′-CCAGTAGAGCGCACAGTATGGT-3′
	Probe	6FAM-5′-CGCACCAGCGGGTGAAGTACACC-3′-TAMRA
*SETDB1*	Forward	5′-GCCGTGACTTCATAGAGGAGTATGT-3′
	Reverse	5′-GCTGGCCACTCTTGAGCAGTA-3′
	Probe	6FAM-5′-TGCCTACCCCAACCGCCCCAT-3′-TAMRA
*GAPDH*	Forward	5′-CGAGATCCCTCCAAAATCAA-3′
	Reverse	5′-TTCACACCCATGACGAACAT-3′
	Probe	6FAM-5′-TCCAACGCAAAGCAATACATGAAC-3′-TAMRA

## Data Availability

The data and research material that support the findings of this study are available from the authors for restrictions [F.A. and A.P.] upon reasonable request (the data are not publicly available due to privacy restrictions).
